# Jaundice Caused by Hyperemesis Gravidarum

**DOI:** 10.31486/toj.22.0019

**Published:** 2022

**Authors:** Angsupat Pornchai, Patarapong Kamalaporn, Chutintorn Sriphrapradang

**Affiliations:** Department of Medicine, Faculty of Medicine Ramathibodi Hospital, Mahidol University, Bangkok, Thailand

**Keywords:** *Hyperemesis gravidarum*, *liver diseases*, *pregnancy*, *pregnancy complications*, *thyrotoxicosis*

## Abstract

**Background:** Hyperemesis gravidarum is characterized by intractable vomiting and associated with weight loss exceeding 5% of prepregnancy body weight, dehydration, and ketosis. Hyperemesis gravidarum occurs during the first trimester and typically resolves by 16 to 20 weeks of gestation. Approximately half of all hospitalized females with hyperemesis gravidarum have a mild elevation in liver enzymes; however, jaundice and hepatic synthetic dysfunction are uncommon.

**Case Report:** A 22-year-old gravida 1 para 0 in her ninth week with a singleton gestation was hospitalized with persistent nausea, vomiting, weight loss of 11% of her prepregnancy body weight, dehydration, hypokalemia, and jaundice. Liver function tests showed hyperbilirubinemia of 7.1 mg/dL and alanine aminotransferase levels high as 676 U/L. Other hepatobiliary diseases were excluded. Thyroid function tests revealed thyrotoxicosis. Gestational thyrotoxicosis is often associated with hyperemesis gravidarum because of their shared pathophysiology of high human chorionic gonadotropin levels during the first trimester. After supportive management including hydration, correction of electrolyte disturbance, vitamin supplementation, and antiemetic treatment, the patient's symptoms resolved. Liver and thyroid dysfunction returned to normal after resolution of vomiting. The patient delivered a healthy child at 38 weeks’ gestation.

**Conclusion:** Elevation of aminotransferase and bilirubin levels may occur in patients with hyperemesis gravidarum. Although jaundice and highly elevated liver enzymes have been reported, investigations to exclude preexisting and concurrent liver diseases are required. Management of hyperemesis gravidarum is supportive, and outcomes are generally favorable.

## INTRODUCTION

Diagnosis and management of liver disease during pregnancy can be challenging for clinicians. Increased liver enzyme levels are sometimes associated with benign disease and favorable outcomes, while some liver diseases can cause serious hepatobiliary dysfunction and result in significant morbidity and mortality for both mother and fetus. The types and presentations of liver disease during pregnancy are varied and can be classified into liver diseases unique to pregnancy and those unrelated to pregnancy. Liver diseases unrelated to pregnancy can be further classified into preexisting diseases that might become active during pregnancy (cirrhosis, portal hypertension, hepatitis B and C, autoimmune liver disease, Budd-Chiari syndrome, and Wilson disease) and diseases or conditions that occur concurrently with pregnancy (viral hepatitis, biliary disease, liver transplantation, and drug-induced hepatotoxicity).^1^

Pregnancy causes significant changes in liver physiologic conditions; these effects can be exacerbated in women with preexisting or concurrent liver disease. For example, females with Budd-Chiari syndrome are at risk of developing an exacerbation during pregnancy because of pregnancy-induced hypercoagulability^2^ and the pressure of the gravid uterus on the inferior vena cava. Gallstones are common in pregnancy because of increased biliary secretion of cholesterol, increased lithogenic bile formation, and decreased gallbladder motility.^3^

Pregnancy-specific liver diseases exhibit a trimester-specific occurrence, while nonrelated liver diseases and conditions can develop at any stage of pregnancy. Hyperemesis gravidarum, a pregnancy-specific liver disease, is severe nausea and vomiting and the most common cause of abnormal liver function in early pregnancy.^4-6^ Elevations in bilirubin and aminotransferases are typically mild to moderate but can be severe. Hyperemesis gravidarum is often accompanied by gestational transient thyrotoxicosis.^7,8^ The evidence shows that 66% to 73% of females with severe hyperemesis gravidarum have elevated thyroid hormone levels.^7,8^ Both hyperemesis gravidarum and elevated thyroid hormone levels are related to elevated serum human chorionic gonadotropin (hCG) hormone levels that rapidly rise in the first trimester. The hCG hormone has weak thyroid-stimulating activity because of considerable homology between the beta-subunit of hCG and thyroid-stimulating hormone (TSH).^9^ In a report of 63 females with high hCG concentrations of >200,000 U/L, TSH was ≤0.2 mU/L in 67% of specimens, and free thyroxine (FT_4_) was >1.8 ng/dL in 32% of specimens. All females with an hCG >400,000 U/L had suppressed TSH.^10^ Very high hCG levels can be found in females with multiple pregnancies (2 or more fetuses) and hyperemesis gravidarum.^11^

Hyperemesis gravidarum usually resolves by 16 to 20 weeks of gestation and generally requires only supportive management.^12^ Outcomes are usually favorable for both mother and offspring if the condition is managed properly. However, abnormal liver biochemical tests that are more marked and persistent than the usual liver dysfunction seen in patients with hyperemesis gravidarum should raise suspicion of an alternative diagnosis such as viral, alcohol, autoimmune, drug, toxin, or biliary pathology.^1^

We report an unusual presentation of jaundice and highly elevated transaminase levels in a patient with hyperemesis gravidarum and gestational transient thyrotoxicosis.

## CASE REPORT

A 22-year-old gravida 1 para 0 female at 9 weeks’ gestation was hospitalized because of severe, protracted nausea and vomiting associated with dehydration for the prior 1 week. She had jaundice for the same duration with no pruritus. She was not able to eat or drink for 1 week before admission. The patient had lost 11% (6.9 kg) of her prepregnancy body weight. Her medical history was unremarkable. She denied smoking, drinking alcohol, and taking any medication, including herbal or over-the-counter medications. Her temperature was 36.3 °C, blood pressure was 105/73 mm Hg, and pulse rate was 117/min. She was jaundiced with anorexia and nausea but had no evidence of hepatosplenomegaly or peripheral stigmata of chronic liver disease. Total bilirubin was 7.1 mg/dL (reference range, 0.2-1.2 mg/dL), direct bilirubin was 5.4 mg/dL (reference range, 0.0-0.5 mg/dL), alanine aminotransferase (ALT) was 676 U/L (reference range, 7-56 U/L), and aspartate aminotransferase (AST) was 349 U/L (reference range, 5-34 U/L) (Table 1). Alkaline phosphatase was within the normal range. International normalized ratio of 1.24 was slightly elevated. Hypokalemia (serum potassium level 3.35 mEq/L [reference range, 3.50-5.10 mEq/L]) was also found. Urinalysis revealed a large amount of ketone. Serum amylase and serum lipase were within the normal range. Serologies for viral hepatitis A, B, C, E, cytomegalovirus, Epstein-Barr virus, herpes, and HIV were negative. There was no evidence for autoimmune hepatitis (negative antinuclear and anti–smooth muscle antibodies).

**Table 1.** Patient's Liver and Thyroid Function Test Results by Week of Gestation

		Week of Gestation							
Variable	Reference Range	9, Admission Day 1	9, Admission Day 4	10	11	12	16	24	28
Aspartate aminotransferase, U/L	5-34	349	128	62	66	26	21	19	22
Alanine aminotransferase, U/L	7-56	676	378	172	91	35	19	16	20
Aspartate aminotransferase/alanine aminotransferase ratio	<1	0.52	0.34	0.36	0.73	1.11	1.11	1.19	1.10
Alkaline phosphatase, U/L	40-150	117	88	79	66	56	53	93	93
Gamma-glutamyl transferase, U/L	9-36	208	135	106	108	61	28	27	27
Total bilirubin, mg/dL	0.2-1.2	7.1	1.9	0.8	1.2	0.6	0.4	0.5	0.4
Direct bilirubin, mg/dL	0.0-0.5	5.4	1.5	0.6	0.8	0.4	0.2	0.2	0.2
Albumin, g/L	35-50	43.2	27.9	32	29.2	33.3	32.4	32.7	31.5
Globulin, g/L	14-48	46.5	28.5	38	30.9	32.3	38.2	40.8	41.6
Cholesterol, mg/dL	<200	132	95	185	167	168	199	280	284
Prothrombin time, s	10.5-13.5	14.5	–	11.8	–	–	–	–	11.6
International normalized ratio	0.91-1.17	1.24	–	0.98	–	–	–	–	0.97
Activated partial thromboplastin time, s	22-33	23.8	–	24.3	–	–	–	–	24.8
Thrombin time, s	14.4-20.8	17	–	–	–	–	–	–	15.3
Total thyroxine, μg/dL	4.87-11.72 (7.31-17.58)^a^	22.5	–	–	–	14.2	14.2	12.8	–
Free thyroxine, ng/dL	0.7-1.48	4.51	–	1.99	–	–	–	–	0.94
Total triiodothyronine, ng/dL	64-152 (96-228)^a^	–	–	–	–	112	112	121	–
Free triiodothyronine, pg/mL	1.88-3.18	5.93	–	4.01	–	–	–	–	2.26
Thyroid-stimulating hormone, mU/L	0.35-4.94	<0.0038	–	<0.0038	–	0.034	0.034	0.754	0.455

^a^The reference ranges for total thyroxine and triiodothyronine levels for patients in the second and third trimester are 1.5-fold higher than in nonpregnant females because of thyroxine-binding globulin excess.

Note: Thyroid function tests were analyzed using the Architect i2000 immunoassay analyzer (Abbott). The analytic sensitivity is 0.4 ng/dL for free thyroxine, 1 μg/dL for total thyroxine, 1 pg/mL for free triiodothyronine, 0.25 ng/dL for total triiodothyronine, and 0.01 mU/L for thyroid-stimulating hormone.

Ultrasonography of the upper abdomen revealed normal liver size with relatively accentuated brightness of the portal vein radical walls (starry sky appearance) that was compatible with acute hepatitis. The gallbladder was partially distended, and the presence of bile sludge was detected. No evidence of common bile duct or intrahepatic duct dilatation was seen. Sonographic appearance of the pancreas and spleen was normal. Obstetric ultrasonography performed to exclude molar pregnancy and multiple gestations demonstrated a normal singleton pregnancy.

FT_4_ was 4.51 ng/dL (reference range, 0.7-1.48 ng/dL), free triiodothyronine (FT_3_) was 5.93 pg/mL (reference range, 1.88-3.18 pg/mL), and TSH was undetectable. The FT_3_/FT_4_ ratio was 1.31 10^−2^pg/ng (a ratio <2.7 10^−2^pg/ng is suggestive of gestational thyrotoxicosis instead of active Graves disease).^13^ However, the patient had no signs or symptoms of thyrotoxicosis. There was no family history of thyroid disease. No thyroid gland enlargement or ophthalmopathy was observed. TSH receptor antibody was negative. Hyperemesis gravidarum and gestational thyrotoxicosis were diagnosed.

The patient's condition improved after 3 days of fluid and electrolyte replacement: intravenous (IV) normal saline with potassium 40 mEq/L at a rate of 100 mL/hour, vitamin B6 100 mg orally twice daily, IV thiamine 100 mg daily for 3 days (for prevention of Wernicke encephalopathy), IV dimenhydrinate 50 mg every 6 hours as needed, and metoclopramide 10 mg orally 3 times daily before meals.

Liver enzymes and serum bilirubin concentrations decreased by day 4 of hospitalization (ALT 378 U/L, AST 128 U/L, total bilirubin 1.9 mg/dL) (Table 1). Serial monitoring of liver and thyroid function tests is shown in the Figure. She was discharged on day 4 of hospitalization. Jaundice resolved at 10 weeks’ gestation.

**Figure.**
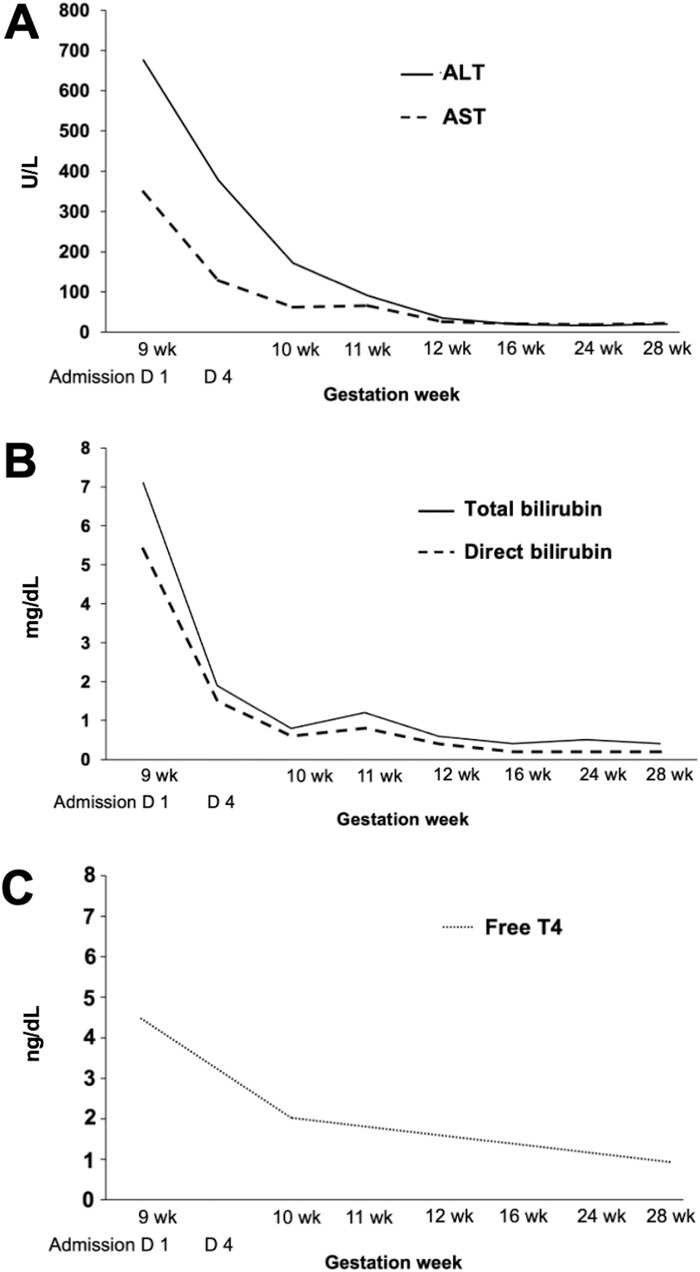
**Serial monitoring by gestation week of (A) alanine aminotransferase (ALT) and aspartate aminotransferase (AST), (B) bilirubin, and (C) free thyroxine (T4).** D1, admission day 1; D4, admission day 4; wk, week.

Three weeks after hospital discharge, the patient remained asymptomatic, and all liver blood tests were normal. Hyperemesis gravidarum disappeared at 14 weeks’ gestation. The pregnancy continued uneventfully, and she delivered a healthy child with a birth weight of 2,700 g at 38 weeks’ gestation.

## DISCUSSION

During pregnancy, the liver biochemical profile remains within the normal range for a nonpregnant state with a few exceptions.^14^ Serum albumin normally decreases as a result of hemodilution, and alkaline phosphatase increases in the third trimester because of placental production and fetal bone development. However, severe liver disease can occur in up to 3% of pregnancies,^15^ and severe pregnancy-related liver diseases can lead to fatal complications in both mother and child.^16,17^

Liver diseases unique to pregnancy include hyperemesis gravidarum, acute fatty liver of pregnancy, intrahepatic cholestasis of pregnancy, and preeclampsia with liver involvement (hemolysis, elevated liver enzymes, and low platelets [HELLP] syndrome). The gestational age of the pregnancy can help in the differential diagnosis. The characteristics of pregnancy-related liver diseases are summarized in Table 2.^1,18,19^

**Table 2.** Characteristics of Pregnancy-Related Liver Diseases^1,18,19^

Characteristic	Hyperemesis Gravidarum	Intrahepatic Cholestasis of Pregnancy	Acute Fatty Liver of Pregnancy	HELLP Syndrome
Trimester	First through 20 weeks	Second/third	Third	Third/early postpartum
Incidence, %	0.3-3	0.3-5.6	0.01	0.2-0.6
Clinical features	Vomiting, dehydration, weight loss, ketosis	Pruritus, jaundice	Abdominal pain, vomiting, polydipsia/polyuria, ascites, encephalopathy, hypoglycemia	Abdominal pain, vomiting, hypertension, proteinuria, headache, seizure, edema
Platelets	Normal	Normal	Decreased	Decreased
Hemolysis	No	No	No	Increased
Bilirubin, mg/dL	<4	<5	<10	<5
Aminotransferases	1 to 2 × ULN	1 to 5 × ULN	5 to 10 × ULN	1 to 100 × ULN
Alkaline phosphatase	Normal	Increased	Increased	Increased
Creatinine	Normal	Normal	Increased	Normal
Proteinuria	No	No	Normal or increased	Normal or increased
Liver imaging	Normal parenchyma without biliary obstruction	Normal parenchyma without biliary obstruction	Fatty infiltration, bright liver	Hepatic infarcts, hematoma, rupture
Complications	Usually resolves by 16-20 weeks without complications	Premature birth, fetal distress, late intrauterine death	High maternal and fetal/perinatal mortality	High maternal and fetal/perinatal mortality
Treatment	Supportive management, rehydration, antiemetics, vitamins	Ursodeoxycholic acid 10-15 mg/kg and early delivery at 37 weeks	Delivery at 34 weeks	Controlled hypertension, correction of coagulopathy, and delivery at 34 weeks; corticoids to promote fetal lung maturity
Recurrence	Very frequent	Very frequent	Rarely	Frequent

Note: This table was modified from García-Romero et al^19^ in accordance with the provisions of Creative Commons license CC BY-NC-ND 4.0.

HELLP, hemolysis, elevated liver enzymes, and low platelets; ULN, upper limit of normal.

Hyperemesis gravidarum is a severe form of nausea and vomiting during pregnancy that results in weight loss of more than 5% of body weight, dehydration, ketonuria, and electrolyte abnormalities. The prevalence of hyperemesis gravidarum is 0.3% to 3% of pregnancies.^20,21^ The pathogenesis of hyperemesis gravidarum is unknown and likely multifactorial, with causes including hormonal changes, abnormal gastrointestinal motility, *Helicobacter pylori* infection, and genetic factors. Serum hCG levels in patients with hyperemesis gravidarum are higher than in other pregnant patients with mild nausea and vomiting.^8,22^ Symptoms of hyperemesis gravidarum are worse in patients with multiple pregnancies and hydatidiform moles, conditions associated with high hCG levels. However, high hCG levels are not consistently associated with the severity of nausea and vomiting.^23^ The presence of specific hCG isoforms or hCG receptor mutations may explain variations of symptoms among patients with similar hCG levels.^24-26^

Symptoms typically start at 5 to 6 weeks of gestation, peak at 9 weeks, and usually resolve by 16 to 20 weeks. Liver enzyme elevations have been reported in 50% to 60% of patients hospitalized with hyperemesis gravidarum.^1^ In the patients with liver enzyme elevations, hyperemesis gravidarum occurred at the 14th week of gestation compared to the 6th week in the normal liver enzyme group. Also, a high degree of ketonuria and the presence of thyrotoxicosis were found in the patients with liver enzyme elevations.^8,27^ Serum aminotransferases are typically mildly elevated to 2 times the upper normal limit.^28^ Values are rarely more than 200 U/L but may be as high as 1,000 U/L.^29-32^ ALT levels are usually greater than AST levels.^5,28,33^ Liver enzyme levels decrease quickly when the vomiting stops.^30^ Rarely, mild jaundice may be seen, and bilirubin can increase to 4 mg/dL.^28^ Elevated serum amylase levels may be seen, but production is from the salivary glands instead of the pancreas.^34^ Previously reported cases of hyperemesis gravidarum with unusual liver function are summarized in Table 3. In one case, a patient experienced 3 episodes of jaundice related to hyperemesis gravidarum in 3 consecutive pregnancies.^29^

**Table 3.** Summary of Hyperemesis Gravidarum Cases With Aspartate Aminotransferase and/or Alanine Aminotransferase Levels >10 Times the Upper Limit of Normal and/or Total Bilirubin >5 mg/dL

Variable	Larrey et al, 1984^29^	Conchillo et al, 2002^30^	Vitoratos et al, 2006^32^	Matsubara et al, 2012^31^	Matsubara et al, 2012^37^	Whitfield et al, 2019^36^	Present case, 2022
Age, years	30	30	35	30	Not reported	25	22
Number of fetuses	Twins	Singleton	Singleton	Singleton	Singleton	Singleton	Singleton
Gestation, weeks	5	14	14	10	12	13	9
Coexisting liver disease/precipitating factor	No	No	No	Drug-induced (folic acid)	No	No	No
Peak aspartate aminotransferase, U/L	22 × upper limit of normal	705	567	732	240	197	349
Peak alanine aminotransferase, U/L		1,674	1,056.4	1,433	518	727	676
Peak total bilirubin, mg/dL	5.96	2.63	6.2	12.9	5.2	7.89	7.1
Ultrasound upper abdomen	Normal	Normal	Normal	Normal	Biliary sludge in the gallbladder	Biliary sludge in the gallbladder	Liver with starry sky appearance; biliary sludge in the gallbladder
Thyroid function	Not reported	Normal	Thyrotoxicosis	Not reported	Not reported	Not reported	Thyrotoxicosis
Liver biopsy	Cholestasis with scarce necrotic hepatocytes in the centrilobular area	Not performed	Not performed	Cholestasis with small necrotic areas within the hepatic lobule	Not performed	Not performed	Not performed
Fetal/neonatal outcomes	Good	Good	Good	Good	Not reported	Not reported	Good

The pathophysiology of hepatocellular injury in hyperemesis gravidarum is not well understood and is probably multifactorial, involving hypovolemia, malnutrition, starvation, lactic acidosis,^27^ and placental tumor necrosis factor alpha.^35^ An association between jaundice in hyperemesis gravidarum and the presence of biliary sludge has been described^36,37^ as occurred in our case. After prolonged fasting, patients can develop bile sludge.^38^ In addition to dehydration, the formation of biliary sludge in pregnancy is attributed to progesterone-induced gallbladder hypomotility^39^ and estrogen-induced changes in bile composition that increase the lithogenicity.^40^

No specific treatment is required for liver dysfunction associated with hyperemesis gravidarum. The mainstay of supportive management is IV rehydration and correction of electrolyte abnormalities (hyponatremia, hypokalemia).^1^ At the initiation of rehydration, thiamine is essential for the prevention of Wernicke encephalopathy. Treatment of nausea and vomiting with first-line pharmacotherapy, vitamin B6 or vitamin B6 plus doxylamine, is effective and safe. Antiemetics such as metoclopramide, promethazine, and ondansetron can be safely used in pregnancy. Dietary modifications should focus on eating small, frequent, low-fat meals and avoiding trigger food items. Enteric nutrition via nasogastric access or parenteral nutrition may be considered in cases of refractory hyperemesis gravidarum. In the setting of hyperemesis gravidarum with gestational thyrotoxicosis, antithyroid drugs are not administered because of the transient nature of the condition; thyroid function normalizes without antithyroid drugs.^41^

## CONCLUSION

Hyperemesis gravidarum can cause mild bilirubinemia and elevated liver enzyme levels. Although jaundice and highly elevated liver enzymes have been reported, investigations to exclude other diseases such as preexisting and concurrent liver diseases are required. Management of hyperemesis gravidarum is supportive, and outcomes are generally favorable.

## Notes

*This article meets the Accreditation Council for Graduate Medical Education and the American Board of Medical Specialties Maintenance of Certification competencies for Patient Care and Medical Knowledge.*
